# Vitamin D Supplementation and Mortality in Older Adults: A Systematic Review and Meta-Analysis

**DOI:** 10.1093/geroni/igaa057.781

**Published:** 2020-12-16

**Authors:** Yunli Zhao, Shuli Jia, Meiling Ge, Birong Dong

**Affiliations:** 1 West China Hospital, Sichuan University, Chengdu, Sichuan, China; 2 West China Hospital, Sichuan University, Chengdu, Shanxi, China; 3 West China Hospital Sichuan University, Chengdu, Sichuan, China

## Abstract

Web of Science, Embase, Medline and Cochrane Central Register of Controlled Trails from their inception to 14 January 2020 were searched in this review. We included randomized controlled trials (RCTs), which compared vitamin D supplementation versus placebo or no intervention and reported mortality as one of outcomes in older adults. Two review authors extracted data independently. Fifty-one articles were included in the analysis, which generated a total pooled sample of 89,977 people and 9813 deaths. These trials were pooled in a meta-analysis, and the outcomes were expressed as risk ratios (RRs) and 95% confidence Intervals (CIs). Across all studies, vitamin D supplementation was not associated with all-cause mortality (RR 0.98, 95%CI 0.92 to 1.04, P=0.48, I2=14%; 89,977 participants; data from 51 trails). Vitamin D supplementation was significantly decreased cancer mortality ( RR 0.85, 95% CI 0.74 to 0.97, P= 0.02, I2=0%; 34364 participants; data from 6 trails). A subgroup analysis showed the associations between the length of vitamin D supplementation more than 3 years and all-cause mortality were statistically significance (RR 0.93, 95%CI 0.88 to 0.98, p = 0.01, I2=0%; 49336 participants; data from 17 trails). Subgroup analyses by vitamin D status, forms of vitamin D (vitamin D3, vitamin D2, alfacalcidol or calcitriol), dose showed no association with all-cause mortality. The evidence from pooled analysis of 51 RCTs undertaken in older adults shows vitamin D supplementation was not associated with all-cause mortality. More long-term trails are need to know weather vitamin D supplementation can decrease all-cause mortality in older adults.

